# Complete genome sequence of the endophytic *Pseudomonas bijieensis* BR-1-Y isolated from the roots of medicinal plant *Stemona japonica*

**DOI:** 10.1128/mra.00070-26

**Published:** 2026-05-18

**Authors:** Jiatong Zhang, La Li, Heqi Dai, Tao Chen, Yusha Du, Kangwei Xie, Xingyong Yang

**Affiliations:** 1 College of Pharmacy, Chengdu University, Chengdu, China; 2 Chongqing Kaizheng Food Co., Ltd., Chongqing, China; 3 Sichuan Quantai Tang Chuan Angelica Dahurica Industry Co., Ltd., Suining, China; California State University San Marcos, San Marcos, California, USA

**Keywords:** *Pseudomonas bijieensis*, *Stemona japonica*, endophyte, genome, medicinal plant

## Abstract

*Pseudomonas bijieensis* BR-1-Y is an endophytic bacterium isolated from the medicinal plant *Stemona japonica* in Chongqing, China. Whole-genome sequencing revealed a single circular chromosome comprising 6,713,544 bp and containing 5,824 protein-coding genes, 65 tRNA genes, and 16 rRNA genes.

## ANNOUNCEMENT

*Pseudomonas bijieensis* is a newly characterized species in the *Pseudomonas* genus (1). However, genomic data on the endophytic *P. bijieensis* remain limited. *P. bijieensis* BR-1-Y was isolated from surface-sterilized roots of *Stemona japonica* (Bai Bu) collected in May 2021 in Sanquan Town, Nanchuan District, Chongqing, China (29°7′58.49″N, 107°12′11.72″E). Plant tissues were sterilized using 75% ethanol and 2% sodium hypochlorite, followed by thorough rinsing with sterile water. The surface-sterilized tissues were then cut into small pieces and directly placed onto LB agar plates (2). Single colonies were purified through repeated streaking. Genomic DNA was extracted from a 48 h LB broth culture grown at 28°C and 180 rpm using the cetyltrimethylammonium bromide (CTAB) method (3).

Sequencing was conducted by Shanghai Personalbio Technology Co., Ltd. Illumina sequencing was conducted using the Illumina HiSeq 4000 platform. Libraries were prepared using the TruSeq DNA Sample Prep Kit, and genomic DNA was fragmented to approximately 400 bp with a Covaris system. Adapter trimming was performed with AdapterRemoval v2.1.7 (4), and sequencing errors were corrected with SOAPec v2.0 (5). Additionally, PacBio long-read sequencing was performed on the PacBio RS platform (PACBIO_SMRT). Genome assembly was carried out using HGAP (v.2021-08) with default parameters (6) and subsequently polished with Pilon v 1.22 (7) utilizing Illumina reads. The Illumina sequencing data provided an average genome coverage of approximately 143.5×.

The assembly resulted in a single circular chromosome of 6,713,544 bp. Genome rotation and overlap trimming were performed based on the dnaA locus. Annotation was conducted using the National Center for Biotechnology Information (NCBI PGAP v6.10) with default parameters. A total of 5,824 protein-coding genes, 65 tRNA genes, and 16 rRNA genes were identified (Table 1).

**TABLE 1:** Genome statistics for *Pseudomonas bijieensis* BR-1-Y

Attribute	Value
Genome size (bp)	6,713,544
Genome topology	Circular
Contigs	1
GC content (%)	60.78
Protein-coding genes (CDSs)	5,824
tRNAs	65
rRNAs (5S/16S/23S)	16 (6/5/5)
ncRNAs	4
CRISPR arrays	2
Illumina reads	3,352,354
Read length (bp)	150
Library layout (paired-end)	2 × 150 bp
PacBio reads	569,267
Reads N50 (bp)	10,928

## Data Availability

This Whole Genome Shotgun project has been deposited in GenBank under accession number JBSEOM000000000. The raw sequencing reads have been deposited in the SRA under the following accession numbers: SRR35651424 for Illumina and SRR35651423 for PacBio. These data are associated with BioProject PRJNA1334109 and BioSample SAMN51830408.
